# Penetration and Toxicity of Nanomaterials in Higher Plants

**DOI:** 10.3390/nano5020851

**Published:** 2015-05-26

**Authors:** Giuseppe Chichiriccò, Anna Poma

**Affiliations:** Department of Life, Health and Environmental Sciences, University of L’Aquila, Via Vetoio, I-67010 Coppito, L’Aquila, Italy; E-Mail: annamariagiuseppina.poma@univaq.it

**Keywords:** carbon nanomaterials (CNMs), carbon nanotubes (CNTs), cytotoxicity, multi-walled carbon nanotubes (MWCNTs), nanomaterials (NMs), nanoparticles (NPs), single-walled carbon nanotubes (SWCNTs)

## Abstract

Nanomaterials (NMs) comprise either inorganic particles consisting of metals, oxides, and salts that exist in nature and may be also produced in the laboratory, or organic particles originating only from the laboratory, having at least one dimension between 1 and 100 nm in size. According to shape, size, surface area, and charge, NMs have different mechanical, chemical, electrical, and optical properties that make them suitable for technological and biomedical applications and thus they are being increasingly produced and modified. Despite their beneficial potential, their use may be hazardous to health owing to the capacity to enter the animal and plant body and interact with cells. Studies on NMs involve technologists, biologists, physicists, chemists, and ecologists, so there are numerous reports that are significantly raising the level of knowledge, especially in the field of nanotechnology; however, many aspects concerning nanobiology remain undiscovered, including the interactions with plant biomolecules. In this review we examine current knowledge on the ways in which NMs penetrate plant organs and interact with cells, with the aim of shedding light on the reactivity of NMs and toxicity to plants. These points are discussed critically to adjust the balance with regard to the risk to the health of the plants as well as providing some suggestions for new studies on this topic.

## 1. Introduction

Plants play a key role in the flow of energy from the sun to other organisms and are thus fundamental for any terrestrial ecosystem. Their cellular lines from gamy (zygote) and from meiosis (spores) develop into diploid sporophytic and aploid gametophytic generations, respectively. Sporophytes have perpetually young tissues at their ends (meristems) to grow organs indefinitely and to develop predominant surfaces to absorb water from the soil and sunlight, and carbon dioxide from the atmosphere. In anthropized environments these surfaces are exposed to pollutants on a daily basis, the aerial ones providing a huge landing field for airborne particles and the subterranean ones a kind of drain for pollutants absorbed by the soil. Among pollutant particles [1], there are nanomaterials (NMs) consisting of nanoparticles (NPs) and carbon-based nanomaterials (CNMs). NPs are particles 1–100 nm in size. CNMs can vary in length from tens of nanometers to a few centimeters and consist of: (1) single layered cylinders, named single-walled carbon nanotubes (SWCNTs), usually not exceeding 2 nm in diameter; (2) multiple concentric layers of cylinders not exceeding 80 nm in diameter and named multi-walled carbon nanotubes (MWCNTs); (3) fullerenes, which are hollow spheres less than 1 nm; and (4) graphene, which is a carbon allotrope made of a single layer of atoms arranged in repeating structures of hexagon shape as honeycomb. Functionalized NMs can be obtained by linking a number of chemical functional groups to their surface [2]. NPs may arise from natural (volcanic eruption), accidental (plants combustion), and planned (engine cars) activities, and both NPs and CNMs are produced in the laboratory by man for their potential use in the fields of agriculture, chemistry, biology, medicine, and energy [3,4]. Nanoparticles (nanogold positively charged) have even been used as useful probes to gain insight into the mechanisms of the plasma membrane internalization (endocytosis) in plant cells [5]. In addition, there is growing interest in the use of surface-functionalized NMs as absorbers of heavy metals from polluted environments [6]. A number of studies draw particular attention to the vulnerability of animals and plants to NMs [7,8,9], but the penetration and translocation in plants and the toxicity mechanisms are still poorly understood. Plant organs are typically protected by coatings and cell walls, which, notwithstanding their sealing capability, may be overcome by NMs. Once these penetrate into the plant, harmful effects may be produced, as well as effects advantageous for man. Here we give an updated survey on the cellular pathways for uptake and translocation of NMs and their effects in higher plants.

## 2. Vegetative System

**Figure 1:**
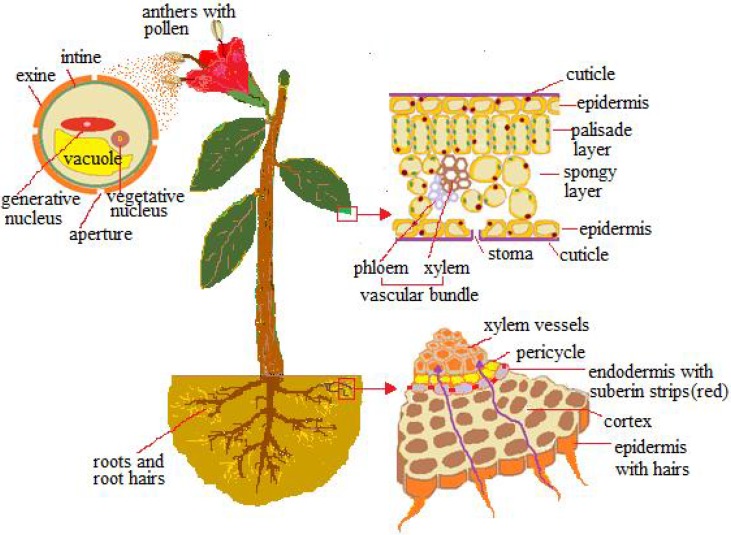
Pathways by which nanomaterials (NMs) are absorbed: pollen exine, cuticle, stomata, and root hairs.

The epidermis foliar (Figure 1) is made up of cells with the exposed walls waterproofed by hydroxylated fatty acids (cutin and waxes), which form a cuticle membrane. Epidermis is provided with stomata ranging from 100 to 1300 per mm^2^, consisting of two guard cells, which, through expansion (turgor), form a pore between them ranging from 3 to 12 µm in width and 10–30 µm in length, for gas exchange. Eichert and Goldbach [10], according to the polar pore model [11], estimated 2–2.4 nm as the exclusion limit of the pore radius for polar and ionic solutes to penetrate the cuticle, while for diffusion via the stomatal surface the pore radius was quite variable and always exceeded 20 nm. The amplitude of the stomatal pores practically usable for the passage is not measurable; it is controlled by a number of factors such as the water layers and bacteria layers, among others.

The pathway for penetrating the cell is through the openings of the primary cell wall, consisting of a polysaccharidic-proteic structure—which, depending on the pectic component, is more or less porous [12]; the pore sizes range from 3.5 to 20 nm [13,14,15] and more often are around 5 nm. The progression to protoplasm (symplastic way) is made possible by cell membrane-embedded protein carriers and ionic channels, or by membrane invagination (endocytosis), which forms vesicles around the passenger. The transport cell to cell is made dynamic by cytoplasmic channels, the plasmodesmata, which are 20–50 nm in diameter at the midpoint and usually let inside small particles, around 3 nm [1], but the exclusion limits are subject to variations as endogenous proteins mediate crossing. Below the foliar epidermis, the photosynthetic palisade tissue (Figure 1) provides small intercellular spaces which, together with the cell walls, form the apoplastic pathway. Through the symplastic and apoplastic pathways, small particles can reach the photosynthate conducting system (phloem vessels), made up of living sieve-cells devoid of a nucleus and most organelles, while being connected to one another and to the surrounding tissues by wall sieve pores of 0.2–0.4 µm.

Underground, the roots are permeable only near the tips, where the epidermal cells extend out into hair roots (Figure 1); the remaining tracts (exodermis) are waterproofed by the suberin. Within the root the cortical apoplastic way is obstructed around the vascular system by a cell layer with radial walls impregnated by suberin (endodermis) (Figure 1), so the path to the vessels is through the tangential walls of the endodermis (symplastic way). However, the lateral roots enable an apoplastic path from the emergence zone up to the vessels [1]. Vessels consist of emptied cellular elements longitudinally connected to one another and to the surrounding cells with pits wider than 1 µm [16,17,18], so it is possible for small particles absorbed with the water to move passively within them.

The stem and its branches are wrapped by a tissue of suberized cells that replaces the epidermis; it is tiered by the radial growth of the stem. The annual and biennial plantlets do not grow radially, so their stems remain enveloped by epidermis provided with stomata.

## 3. Uptake and Toxicity of NMs

Many plants have been tested for their vulnerability to NMs, although only a few of them have been studied extensively with regard to their uptake and pathways for diffusion and internalization. For an exhaustive record, see the Tables in [8].

### 3.1. Leaf

When considering pollution, the first thing that comes to mind is the landing of particles on the leaves followed by penetration and/or obstruction of the stomatal pores, neglecting the potentiality of the particles to traverse the epidermal cuticle. To evaluate this, it must be kept in mind that in horizontally oriented leaves the stomata are usually in the epidermis of the dorsal surface, where airborne particles can scarcely be accumulated.

In *Vicia faba*, Eichert *et al.* [19] observed with confocal microscopy that polymeric NPs (suspensions of modified polystyrene particles) 43 nm in diameter penetrated the stomatal leaf pores, even if only sporadically and solely through a part of the total stomata, while particles of 1.1 μm did not ever penetrate. These observations, which are in agreement with previous studies cited above [10], made it possible to consider a size of 43 nm as the size exclusion limit predictable for the stomata penetration by nanoparticles. Birbaum *et al.* [20] used mass spectrometry and electronic and confocal microscopy and found that CeO_2_–NPs with an average size of 37 nm when administered as spray (total exposure 0.4 g NPs) or in solution (10 ppm NPs) were retained by the leaves of *Zea mays* (50 µg of cerium per gram of leaf) with no sign of translocation to the stem, but the contribution of the stomata and the possible adsorption or incorporation of NPs was not ascertained. Kurepa *et al.* [21] treated seedlings of *Arabidopsis thaliana* grown on agar medium with TiO_2_–nanoconjugates (2.8 ± 1.4 nm), and by using electron and X-ray fluorescence microscopy reported a penetrating ability of NPs into the epidermis and underlying palisade tissue, which suggested a contribution of the stomata and endocytotic vesicles in the absorption. Further studies by means of mass spectroscopy and electron microscopy analysis provided evidence of the foliar uptake following aerial treatments. Watermelon plants grown in pots and having large stomata and vessels were used by Wang *et al.* [22] for spraying with NPs (Fe_2_O_3_, TiO_2_, MgO, ZnO); these initially were 27.3–46.7 nm in diameter and increased considerably in the suspension but significantly reduced during the spray treatment. Particles that did not exceed the diameter of 100 nm penetrated the foliar stomata and were translocated from leaves to stems and roots through the sieve elements. Taran *et al.* [23] used non-ionic colloidal solutions of NPs (Fe, Zn and Mn) in winter wheat to test their concentration in seedlings either arising from pretreated seeds, or sprayed with NPs after growth. They provided evidence for the absorption of Mn and Zn from the foliar epidermis and for translocation of NPs in seedlings that were pretreated. Cucumber plants hydroponically cultured were aerially treated by Hong *et al.* [24] with CeO_2_–NPs of 8 nm ± 1nm (primary diameter) and of 231 nm ± 16 nm (hydrodynamic diameter) either in the form of powder or in suspension. Nanoparticles in both forms penetrated the foliar epidermis, but only the powder treatments succeeded in translocating to stems and roots. Larue *et al.* [25] sprayed the leaves of *Lactuga* with the salt AgNO_3_, and with Ag–NPs, which were both round (38.6 nm in diameter) and non-round (38.2 nm × 57.8 nm) and had hydrodynamic diameters of 47.9 nm ± 29.2 nm. They provided detailed evidence for the cuticolar and stomatal uptake of NPs and translocation up into the vascular tissue through pathways, which seemed to be both apoplastic and symplastic. Moreover, they suggested transformation cycles within the plant involving the binding of Ag^+^ ions to thiol groups and the conversion of Ag^+^ ions in Ag–NPs, starting from dissolution of both the salt AgNO_3_ and Ag–NPs. Larue *et al.* [26] had previously described leaf penetration by TiO_2_–NPs in wheat and rapeseed.

Carbon-based nanomaterials (CNMs) have potential properties for penetration through leaves and translocation to the roots [27]; their foliar uptake is, however, not well documented. Studies on leaf cells are prevalently based on *in vitro* cultures of cells enzymatically treated for removing walls (protoplasts) before culturing, and describe membrane penetration via endocytosis, as reported by Shen *et al.* [28] in *Oryza* and *Arabidopsis* (see also in the following section). Such a penetration pathway was first reported by Liu *et al.* [29] in suspension cultures of intact tobacco cells line BY-2 (from seedling callus) treated with single-walled carbon nanotubes (SWCNTs, length <500 nm) bound to FITC dye for observations with confocal microscopy. Recently Gilardo *et al.* [30], by applying advanced methods to the leaves and to excised chloroplasts of spinach, proved the ability of SWCNTs to cross the stomata foliar as well as to penetrate chloroplasts and accumulate on thylakoids and stroma.

#### 3.1.1. Toxicity of Metal- and Metal Oxide-NPs

The vulnerability of the foliar epidermis allowed for the estimation of the NPs’ effects on the photosynthetic activity of chloroplasts. In *Spinacia oleracea*, Hong *et al.* [31] observed that treatment with TiO_2_–NPs activated a photochemical reaction, and subsequent studies [8] suggested a mechanism based on the penetration of the TiO_2_–NPs into the chloroplast, followed by binding to the photosystem II and activation of the basic reaction—that is, the charge separation. On the contrary, TiO_2_–NPs (2.8 nm ± 1.4 nm) produced microtubule disorganization in epidermal and stomatal cells of wheat seedling leaves [32]. The influence of NPs in the magnetic form was studied by Rãcuciu and Creangã [33] by growing plantlets of *Zea mays* arising from seeds in a culture medium supplemented with magnetite (Fe_3_O_4_–NPs) particles of 8 nm in size, as a ferrofluid suspension. The plantlet growth and the levels of the chlorophylls a, chlorophylls b and carotenoids were stimulated by 10–50 µL/L of Fe_3_O_4_–NPs and inhibited by higher concentrations, while the chlorophylls a/b ratio and the photosynthesis process were decreased either by low or high concentrations of NPs. These results highlighted the potentiality of Fe_3_O_4_–NPs to affect the photosynthetic machinery, suggesting a role of the iron oxides (from magnetite) in the photosynthesis malfunction, and probable interference with the plasma membrane ion channels. Some years later [34] Wang *et al.* observed that Fe_3_O_4_–NPs induced oxidative stress in the shoots and roots of *Lolium perenne* and *Cucurbita mixta*, and to a greater extent than in treatment with Fe_3_O_4_ bulk particles, despite the fact that X-ray absorption spectroscopic analyses excluded internalization of the Fe_3_O_4_–NPs. Parsons *et al.* [35], in leaves of hydroponic seedlings of mesquite treated with 0.10 g Ni(OH)_2_–NPs, found 400–803 mg/kg dry weight of Ni, with no effect on chlorophyll production.

#### 3.1.2. Toxicity of CNMs

Carbon-based nanomaterials (CNMs), owing to their numerous potential applications, have been increasingly used for phytotoxic tests since their ability to enter the cell wall and plasma membrane was ascertained [29]. Studies on leaf cell cultures in *Arabidopsis* [36] and O*ryza sativa* [37] showed that treatments with multi-walled carbon nanotubes (MWCNTs) produced in the first plant a decrease of superoxidedismutase (SOD) activity associated with a decay of chlorophyll production, and in the second plant an increase of reactive oxygen species (ROS) and apoptotic processes. It is notable that symptoms of oxidative stress (ROS accumulation) and a dose-dependent programmed cell death were found in the above plant species by Shen *et al.* [28] after treating both protoplasts and integral leaves with SWNTs; these authors also provided some evidence in favor of the internalization of nanotubes through endocytosis-like processes. Recently, Giraldo *et al.* [30] found that SWCNTs, once having penetrated the membranes of spinach chloroplasts, increased the flow of electrons and photosynthetic activity, most likely by a stimulating action on the uptake of light with wavelengths of the near-infrared. Moreover, SWCNTs were shown to be sensitive to nitric oxides (NOx), suggesting that plants enclosing nanotubes could be used as detectors for NOx. Otherwise, Santos *et al.* [38] showed a toxic action of Fullerene C60 on the aquatic plant *Lemna gibba*, which manifested itself in a decrease of photosynthetic activity and plant growth.

The leaves were also shown to be sensitive to graphene oxides (GO). Cabbage, spinach, and tomato leaves decreased in size when the seedlings were treated with GO at 500–2000 mg/L, and the leaves of tomato and cabbage also decreased in number at concentrations of 1000–2000 mg/L [39].

### 3.2. Roots

Most data come from experiments on young plants developing from seeds, so the seed coat absorbability and the effects of NMs on germination were also proved. The studies on wheat, maize, spinach, zucchini, rapeseed, and some desert plants [23,40,41,42,43,44,45] showed the ability of metal-NPs to penetrate seeds without affecting germination, and some [23] reported the NPs’ distribution in the corresponding seedlings. However, in *Arabidopsis thaliana* the TiO_2_–nanoconjugates smaller than 5 nm remained stuck to the seed mucilage and failed to penetrate [21].

Many plant species are known to absorb NPs by the roots and translocate them in stems and leaves [46], depending on the physicochemical features of NPs, the type of plant, and the growth medium. Experiments in hydroponic maize cultures [15] pointed out that colloidal suspensions of TiO_2_–NPs (1 g/L) with sizes too large (30 nm) for the rhyzoderm cell wall pores (6.6 nm) prejudiced the hydraulic conductivity of the primary roots by a physical action on the wall pores that affected the transpiration and growth of the leaves. The same plants grown in pots and irrigated with TiO_2_–NPs at 1 g/L concentration suffered much less from the treatment. Magnetite nanoparticles of 20 nm (Fe_3_O_4_–NPs) were able to penetrate roots and translocate to leaves of pumpkin plants grown in an aqueous medium [47]. In agar-medium cultures of *Arabidopsis thaliana* seedlings the TiO_2_–nanoconjugates of 2.8 ± 1.4 nm in diameter succeeded in root cell penetration up to inside vacuoles and the nucleus [21]. Likewise, Ni(OH)_2_–NPs, both uncoated (8.7 nm) and coated (2.5 nm before and 0.9 nm after synthesis), penetrated roots in hydroponic seedlings of mesquite and were transformed into an Ni(II)–organic acid complex, which moved to the leaves [35]. Experiments of Zhang *et al.* [48] in cucumber evidenced root absorption of CeO_2_–NPs with hydrodynamic diameters of 40.2 nm ± 7.2 nm in deionized water, and 691.7 nm ± 26 nm in the nutrient solution.

Some data are available on treatments in soil. Du *et al.* [49] treated wheat plants with TiO_2_–NPs and ZnO–NPs (20–100 nm in diameter) under field conditions using outdoor lysimeters. They observed with TEM that TiO_2_–NPs either agglomerated and adhered to the cell walls of the root periderm, or particles of 50 ± 10 nm, probably derived from those of 20 ± 5, penetrated the primary epidermis roots and the cortex via apoplast up into cell vacuoles, whereas ZnO–NPs seemed to dissolve and then penetrate the cells in the form of Zn^2+^ ions. In *Zea mays* [20], no evidence was found for root absorption from soil irrigated with solutions of CeO_2_–NPs having a 37 nm average size. Other experiments using ICP-OES Spectroscopy, X-ray fluorescence, and confocal microscopy reported that the CeO_2_–NPs with primary diameter 8 nm ± 1 nm and hydrodynamic diameter 1373 nm ± 32 nm, when added to the soil where corn plants were growing, penetrated the roots and translocated in the shoots along a path that seemed to be apoplastic [50,51]. The studies also pointed out that organic substances in the soil influenced the mobility of NPs, and alginates favored nanoparticle accumulation in the roots and translocation to shoots.

For other data on the root uptake and sizes of NPs, see the next section.

Carbon nanotubes (CNTs) may either accumulate on the ryzoderm surface or penetrate its cell walls and, if they are compatible with the size, they may be translocated in aerial plant tissues or get trapped during translocation. Accumulation of MWCNTs and SWCNTs was observed on the root surface of several crop plants including tomato, wheat, cabbage, lettuce, carrot, cucumber, onion, and rice [52,53,54,55]. Canas *et al.* [52] observed in several plants that CNTs did not penetrate the roots and produced effects on the root growth—adverse in tomato and advantageous in onion and cucumber—whose mechanisms of action were not fully clarified. At variance was the reported ability of CNTs to produce pores on the tomato seed coat that favored water uptake as well as seed germination and growth of seedlings [56]. Assuming CNTs are able to induce new water entry points on the seed coat, one would expect a similar capacity on the root rhyzoderm instead of a suppression of water uptake. This point, repeatedly debated by several authors, is still waiting to be cleared up.

Evidence for the absorption of carbon-based nanomaterials (CNMs) and translocation to aerial parts, including fruits, were provided by Smirnova *et al.* [57] in *Onobrychis arenaria* seedlings with electron microscopy (TEM), and by Khodakovskaya *et al.* [53] in tomato seedlings with TEM, Raman photothermal, and photoacoustic methods. The first authors used cylindrical MWCNTs 2 µm long with an external diameter of 20–70 nm. The second used: (1) MWCNTs with diameter 10–35 nm and length of 6 µm; (2) SWCNTs with diameter 0.86–2.22 nm and length of a few microns; and (3) graphene with a structure of 2–5 nm in thickness and a diameter of 100–120 nm.

Magnetic carbon-coated nanoparticles ranging from 5 to 50 nm with an average hydrodynamic diameter of 200 nm were used to treat plantlets of sunflower, tomato, pea, and wheat [58]. They were absorbed by the roots and then translocated to the xylem vessels up to the leaves (in wheat up to the trichomes), with a different efficiency depending on the species.

Lin *et al.* [55] after treating rice seeds and plants with CNMs suspended in natural organic matter, found a negligible uptake of MWCNTs, while fullerene C_70_ was found in seeds, seedlings, and the adult plants. Initially the fullerene was present more in the seeds and seedling roots than in the stem and leaves, while in adult plants it was only present neighboring the vascular system and leaves, suggesting uptake and substantial translocation within the plant. Recently, studies on bitter melon [59] substantiated, by Fourier Transform Infrared Spectroscopy (FTIR) analysis, the capacity of fullerol, a fullerene derivative, to penetrate roots and translocate to all parts of the plant including flowers and fruits. The smallest diameters of fullerol were 1.5–5.0 nm; they increased notably by increasing the concentration.

The mechanisms for CNMs’ internalization into the plant are poorly understood, but some knowledge comes from the following studies. In *Catharanthus* plants, Liu *et al.* [29] found that SWCNTs labeled with fluorescein isothiocyanate were able to cross cell walls and the cell membrane through endocytosis, and then penetrate into the vacuole. For this ability, nanotubes have been considered as potential carriers of chemical substances through the cell and its substructures. Later, studies [28] in deprived wall cells (protoplasts) of rice and *Arabidopsis* described the formation of endocytosis-like structures after treatment with SWCNTs. More recent studies in *Nicotiana* and *Catharanthus* [60,61,62,63], in addition to confirming the endocytotic method of the nanotubes, reported their ability to penetrate the nucleus, plastids, and vacuoles, and to induce organelle recycling. In *Catharanthus* cell cultures treated with SWCNTs of 4.5 nm × 0.15–1.5 nm Serag *et al.* [63] by ICP Scanning Raster Image Correlation Spectroscopy, found cytoplasmic material and SWNTs were both incorporated in the vacuole, which was interpreted as a mechanism of autophagy induced by damage to the cytoplasm.

Graphene oxides (GO) may be absorbed by roots. Studies on seeds and seedlings of *Arabidopsis thaliana* treated with GO of sizes 40–60 nm before and of 192 nm ± 24 nm after inclusion in the nutritive fluid, and a thickness of about 1.0 nm, were recently reported [64]. The authors confirmed the absorption of graphene by the roots and pointed out its accumulation in the air roots, rhyzoderma, and parechima root cells, with no evident signs of translocation except to the cotyledon cells.

#### 3.2.1. Toxicity of Metal- and Metal Oxide-NPs

NPs can explicate a cytotoxic action on roots either after penetrating its cells and their organelles, or by inducing chemical changes in the surrounding medium or, more simply, by hindering the absorbing function of the roots.

Yang and Watts [65] treated plants of *Zea mays*, *Cucumis sativus*, *Brassica oleracea*, *Glycine max*, and *Daucus carota* with Al_2_O_3_–NPs and observed reduced root elongation due to toxic action related to the surface characteristics of the particles. Root degrowth had been previously reported in other plant species treated with low concentrations (2 mg/L) of Al_2_O_3_ in the form of phenanthrene-coated NPs [66]. Lin and Xing [67] treated hydroponic cultures of *Lolium perenne* with both Zn^2+^ ions and ZnO–NPs and found that ZnO–NPs were able to cross root cell walls, penetrate the cells, and reach vascular tissue via the endodermis, but the translocation to shoots was limited and much lower than that of Zn^2+^. The uptake damaged the epidermal and cortical cells and decreased the plant biomass, with major effects caused by the Zn ions with respect to ZnO–NPs treatment. The same authors previously reported in other plant species a toxic effect of ZnO–NPs on root growth by using high concentrations (2000 mg/L) [68]. In hydroponic zucchini cultures, Stampoulis *et al.* [42], by using metal-oxide-NPs solutions and their corresponding bulk materials, found that Ag (size 100 nm) and Cu (size 50 nm) were toxic only in the form of nanoparticles, significantly reducing the biomass and plant transpiration as compared with their corresponding controls and bulk materials. The toxic effect as determined for Ag at concentrations of 100–1000 mg/L was dose dependent; it was in part related to the release of Ag ions from NPs and in part to a direct interaction of NPs with cells. The level of Ag within shoot cells, assayed with Inductively Coupled Plasma–Mass Spectrometry (ICP-MS), was much higher after Ag–NPs exposure than after bulk exposure.

Some experiments were carried out using soil or sand as medium. Du *et al.* [49] added TiO_2_–NPs and ZnO–NPs (predominant sizes 20 nm ± 5 nm and 40 nm ± 10 nm respectively) into the soil where wheat plants grew, and observed with TEM that both NPs decreased the activity of proteases, catalase, and peroxidase enzymes in the soil, but only the small TiO_2_–NPs (20 nm) penetrated the rhyzoderm of the primary roots and cortex cells up into vacuoles. The ZnO–NPs were not found in the roots, probably due to the dissolution and release of Zn^+^ ions that had penetrated the root. All these observations were correlated with the loss of plant biomass. Dimkpa *et al.* [69] used ZnO–NPs and CuO–NPs in sand-grown wheat plants and observed accumulation in the shoots of CuO, Cu-sulfur complex, and Zn-phosphate, which induced oxidative stress (ROS increase) with inhibition of root growth. Transformations of ZnO–NPs had been previously reported in other species including some desert plants [43], where seed treatment with ZnO–NPs (8 nm) did not affect germination but the seedling roots showed a trend of reduced length and to transform the NPs into Zn-nitrate, Zn-phosphate, and Zn-citrate. Recently Kouhi *et al.* [45] evaluated in rapeseed seedlings the toxicity of: (1) ZnO–NPs <50 nm; (2) ZnO–microparticles (MPs); and (3) Zn^2+^, and found that Zn^2+^ inhibited root elongation more than MPs and the MPs more than NPs. However, at high concentrations (250, 500 mg/L) the three different treatments had the same toxic effects. The authors hypothesized that the dissolution of the different forms of Zn in contact with the roots played a key role in the toxicity. Further studies on hydroponic maize plants with ZnO–NPs and Zn^2+^ ions [70] suggested that both the Zn^2+^ added in the medium and the Zn^2+^ ions deriving from dissolution of NPs, as well as the NPs that escaped dissolution, entered the roots and were precipitated and entrapped in the form of phosphate, so only a few of the NPs reached the vascular tissue.

Well known for its trend in the ionic form to be biotransformed into nanoparticles is silver [25,71,72]; its biosynthesis finds application in various fields [73]. Treatment with Ag–NPs in the grass *Lolium multiflorum* [74] produced silver accumulation in the roots and in the shoots. This was higher than in treatment with the salt AgNO_3_, which damaged the epidermal and cortical root cells and inhibited the seedlings’ growth with a greater effect when using NPs of 6 nm as compared with those of higher sizes. The treatment with AgNO_3_ or supernatants of the ultracentrifuged Ag–NPs solutions produced no damage. The inverse relationship between the toxicity of Ag–NPs and their size was subsequently observed in *Vicia faba* seedlings [75]. However, in *Zea mays* and *Brassica oleracea* var. *capitata* [44] the treatment with Ag–NPs and ZnO–NPs (hydrodynamic diameters 11 nm ± 0.7 nm) affected the seed germination, root meristem, and root development with toxic effects smaller than those produced by the corresponding ion salts. The above different responses suggested that: (1) Ag–NPs made Ag ions available, especially when in colloidal form; (2) Ag–NPs and Ag ions had a differential absorption by the cell; and (3) Ag–NPs and the corresponding ions operated with different mechanisms of action.

Biogenic formation of metal–NPs was also reported for gold in alfalfa [76] and poplar [77] plants, and for platinum in mustard [78]. In poplar the gold in the form of Au^3+^ ions was absorbed by the roots and transformed into Au–NPs, and in the form of Au–NPs was absorbed without dissolving in Au^3+^ ions. From the roots, Au–NPs moved inside the vascular system to leaves and in the cells of both organs they were in the plasmodesmata, in the cytoplasm, and inside organelles. As a result, the cells were damaged in contrast to what had previously been observed in *Brassica juncea* seedlings [79], where the uptake of Au–NPs following the treatment spray promoted growth and seed yield.

Some metal–NPs considered to be stable, such as CeO_2_–NPs, make plants susceptible to their accumulation, with different effects. In tomatoes Wang *et al.* [80] reported that relatively low concentration (0.1–1 mg/L of CeO_2_–NPs accumulated in the stem, leaves, and fruits without negative effects, and no toxic effects were observed in wheat and pumpkin by either Schwabe *et al.* [81], or Zhao *et al.* [50], despite the fact that nanoparticles were successfully absorbed by the roots. Conversely, Rico *et al.* [82] observed that rice grains from plants grown in soil treated with CeO_2_–NPs reduced their nutritional value as regards minerals, fatty acids, proteins, starch, and antioxidants, some of which are a possible consequence of altered gene expression. Moreover, Zhang *et al.* [48], in hydroponic cultures of cucumber and soybeans treated with CeO_2_–NPs, reported absorption and transformation of cerium. This partly accumulated as CePO_4_ precipitates in the roots because of phosphate availability, and partly translocated in the stem and leaves where they became Ce-carboxylases. Subsequently, Zhang *et al.* [83] found that in *Lactuga* treatment with CeO_2_–NPs, especially when using the smaller NPs (7 nm), increased the levels of ROS, the peroxidation products (MDA), and the antioxidants (SOD, POD), and these were associated with cell death and inhibition of root growth. The toxic effects were attributed to the transformation of Ce from 4+ to 3+ with Ce^3+^ ions release.

Other NPs were reported to change the formula of the core after their absorption, such as La_2_O_3_ to LaPO_4_ in *Cucumis sativus*, Ni(OH)_2_ to Ni^2+^ in *Prosopis* sp. and to Ni(II)-organic acid complex in mesquite, but without affecting the plants [8,35]. However, the dissolution of La_2_O_3_–NPs and Yb_2_O_3_–NPs by secretion of the plant roots made the treatments toxic [83,84].

With regard to the interactions of metal–NPs with root cell substructures, few reports are available. In *Arabidopsis* the accumulation of TiO_2_–nanoconjugates into subcellular compartments did not cause evident damage, also suggesting their application as carriers of short oligonucleotides or peptides into the nucleus [21]. Conversely, in the same plant species treatment with TiO_2_–NPs produced disorganization of the microtubules and isotropic growth of epidermal cell roots, which were ascribed to either the physic interaction of NPs with tubulin or the indirect effect through increasing ROS [32]. Oxidative stress induced by TiO_2_–NPs had also been previously observed in the root of *Allium* [85].

Fluorescent NMs associated with quantum dots (QDs), largely used in animals, were applied in a number of plants [86], and more recently Navarro *et al.* [87] reported for CdSe/ZnS–QDs the induction of oxidative stress in *Arabidopsis* plants.

#### 3.2.2. Genotoxicity of NPs

Tissues mitotically active (root meristem) of several plant species were analyzed for cytogenetic abnormalities induced by NPs. Roots of *Allium cepa*, *Glycine max*, and *Nicotiana tabacum* were exposed to: (1) Ag–NPs; (2) ZnO–NPs and CeO_2_–NPs; and (3) TiO_2_–NPs, for cytological analyses [88], assays with random amplified polymorphic DNA [89], and tests with comet and DNA laddering techniques [85], respectively. The root growth inhibition in the treated plants was associated with typical errors in cell division and chromosome behavior such as bridges, early chromosome separation, multiple breaks, and micronuclei release, as well as DNA damage. Similar mitotic aberrations and DNA alterations were also observed in *Zea mays* and *Vicia narbonensis* treated with TiO_2_–NPs [90], as well as in *Vicia faba* seedlings treated with Ag–NPs [75]. The same cytogenetical changes were previously described by Rãcuciu and Creangã [41] in the root meristem of *Zea mays* treated with magnetic NPs. Oxidative damages to DNA were observed by Atha *et al.* [91] in seedlings of radish and ryegrass after treatment with CuO–NPs and the effects were evident also at low NPs suspension concentrations (10 mg/L).

#### 3.2.3. Toxicity of CNMs

Studies reported advantageous, disadvantageous, or no evident effects of nanotubes on plants both at phenothypic and genotypic levels. A stimulative effect, prevalently of MWCNTs, on seedling root elongation and/or seed germination was observed in onion and cucumber [52], wheat [92], mustard [93], and tomatoes [53,56,94,95]. In tomatoes the MWCNTs penetrated the seed coat and seedling roots through new wall pores and enhanced the capacity of the plant to uptake water, with positive effect on germination and seedling growth. At concentrations of 50–200 µg/mL they improved the vegetative and the reproductive plant development up to double the rate of flowering and fruiting. The stimulation of root emergence and root growth was recently observed in *in vitro* cultures of *Rubus adenotrichos* treated with functionalized SWCNTs [96].

Concerning fullerene and its derivatives, Kole *et al.* [59] observed that in bitter melon the uptake of fullerol (water-soluble) by the roots and its translocation via the stem to leaves, flowers, and fruits, enhanced the plant biomass yield by 54%, its water content by 24%, and the fruit yield by 128%, and even increased the rate of the medical use of components (anticancer) in the fruit. This study also confirmed the transmission of CNMs to the next generation coming from the treated seeds; such a capacity had previously been reported by Lin *et al.* [55] in seeds and plants of rice in the first and second generations, and in these plants the concentration of fullerene (C_70_) was higher in the aerial parts than in the roots, in agreement with its translocation.

No appreciable effect of MWCNTs on seed germination was observed in various crop plants (corn, cucumber, radish, rape, lettuce) [68].

As regards the inhibitory effect, Canas *et al.* [52] observed inhibition of the root growth in tomato and lettuce, although the same treatment on onion and cucumbers stimulated growth. These effects were due to interactions with the root surface as CNTs were not found within the root. As regards graphene, the absorption of GO (0.5–5.0 nm) from the roots of *Vicia faba* seedlings had both beneficial and toxic effects depending on the concentrations; toxic concentrations were of the order of 1600 mg/L and induced oxidative stress and an increase of electrolyte leakage to the detriment of the seedling growth [97]. In suspension cultures of T87 cell line obtained from seedlings of *Arabidopsis thaliana*, GO penetrated cells through endocytotic pathways and at low concentrations not exceeding 80 mg/L induced oxidative stress and damages to the nucleus and mitochondria, which produced cell death [98]. Similar adverse effects had been observed previously in the roots of cabbage and tomato seedlings by using GO at 500–2000 mg/L [39]. Begum and Fugetsu [99] treated spinach plants with MWCNTs and these were observed within the plant tissues to produce an increase of ROS and mechanisms of cell death. The toxicity was ascribed to oxidative stress induced by MWCNTs, as the toxic effects were reversed by adding antioxidants to the MWCNTs treatments.

Interesting results were recently reported by Hu *et al.* [100] in wheat plants after combined treatments of GO and Arsenic. GO and As administered alone were not toxic, but if they were given together, even at a concentration of 0.1 mg/L, they induced oxidative stress and alterations in cellular metabolisms (carbohydrates, amino acids, secondary metabolites) and cell structures, most likely because GO exalted the cell permeability to As, which could therefore damage the cells.

#### 3.2.4. Genotoxicity of CNTs

The genotoxicity of CNTs, referred to as any interaction with the gene expression, has been studied in a few plants. In tomato seedlings Khodakovskaya *et al.* [53], by means of advanced physical methods (Raman, Phototherma, and Photoacoustic methods), described a nanotube-dependent activation, both in roots and leaves, of either stress-related genes, among which were those switched on by pathogens, or the gene controlling the water channels on the plasma membranes. Seeds and seedlings responded to the gene upregulation by increasing germination and growth. Growth induced by the upregulation of genes controlling the cell division, cell wall formation, and the water flow through the membrane was also observed in tobacco cells cultured with MWCNTs [101]. In roots of *Allium cepa*, Ghosh *et al.* [102] observed cytological errors and damages to DNA associated with an apoptosis process, as a probable consequence of internalization of the nanotubes. Most recently Yan *et al.* [103], by means of PCR analysis, immunostaining technique, and electron microscopy, provided evidence that in *Zea mays* the penetration and accumulation of SWCNTs inside roots may change the expression of genes controlling the seminal root and the hairs’ root growth, of benefit to the first and disadvantage to the second.

## 4. Reproductive Systems

Both female and male reproductive generations (gametophytes) of higher plants are exposed with their coats and secretions to the reception and absorption of airborne particles. They are so small as to develop male (pollen) inside the pollen sacs, which open at maturity, and for the female (the primary endosperm or embryo sac) inside the egg, which opens apically with a canal. Angiosperm ovules are inside the pistil, which consists of the ovary and an elongated secretory trait, often channeled, which enlarges at the tip (the stigma) for receiving pollen. Gymnosperms lack pistils and have cones with naked ovules that, by means of a drop of secretion, capture pollen grains and lead them inside as along with any other airborne particle of a few microns in size. Pollen (Figure 1) encloses its cells with an inner wall and an outer wall that is reduced or absent in some dots (pores) or tracts (furrows) [104] to facilitate extrusion of the tube that transports the sperms to ovules. Lipids, proteins, and carbohydrates are spread on the pollen surface for interacting with secretions of the female structures.

## 5. Penetration and Toxicity of Metal- and Metal Oxide-NPs

The studies dealing with the vulnerability of the reproductive systems to NPs are limited to a few reports [105,106]; these, however, are rich in data. These authors studied the effects of Pd–NPs [105] and Ag–NPs [106] on *in vitro* cultured kiwi pollen by using electron microscopy, the SIMAA 6000 instrument for determining palladium, the Atomic Absorption spectrophotometer (AAanalyst 300) for silver, and X-ray resonance fluorescence analysis for the calcium ion Ca^2+^. Both NPs were able to damage the plasma membrane and deplete endogenous calcium, with the result of decreasing the capacity of pollen for germination and elongation with the tube. Pd–NPs penetrated pollen faster and more deeply than PdCl_2_. Ag–NPs increased the levels of ROS, differently to the silver free ion, which was more active than Ag–NPs in damaging the plasma membrane and preventing germination.

A recent study showed that tomato plants treated with relatively low concentrations of CeO_2_–NPs produced, through sexual reproduction, poorly developing progeny with evident signs of oxidative stress and an enhanced capacity to accumulate Ceria [107].

These data are somewhat worrisome, substantiating the presumptive vulnerability of pollen based on discontinuous wall exine and intense metabolic activity concentrated in the vegetative cell producing pollen tube, and even showing transgenerational effects consequential to treatments with NMs.

## 6. Conclusions and Perspectives

The receptivity of plants to NMs goes beyond the predictable one based on their structural traits, as well as the vessels and sieve elements for displacement of NMs upwards and downwards. NMs can overcome the barrier cuticle and then are able to penetrate the pores of the wall that are less selective than the cutin so as to allow the passage of NMs larger than the pores themselves [49,51,108]. Across the cuticle there are two alternative paths of diffusion, the lipophilic path and the polar path, made up of aqueous narrow pores (0.5–2 nm in diameter) whose length and sinuosity are unknown [10,109,110]. How the NMs pass through these paths is yet to be discovered. Across the cell membrane the NMs pass either by diffusion, via carriers, *de novo* formed membrane pores, or by energy-dependent pathways (endocytosis), as well as by linking to chemicals for cell internalization, so mimicking the behavior of biological compounds. It is more than likely that NMs can activate and dynamize processes by which cells become permeable; after all, it is well known that cell substructures may amplify their function when opportunely stimulated, *i.e.*, when the plasmodesmata are in contact with some viral particles [111]. From this perspective, the NMs might open the door to environmental toxicants by carrying them into cells. This hypothesis is substantiated by the increased uptake of pesticides observed in plants (zucchini, tomatoes, and soybeans) treated with C60 [112,113], as well as by recent studies [100] on combined treatments of graphene and arsenic, which suggest a role for graphene in the transport of As into cells.

Within cells the NMs may interact with the molecules structurally and non-structurally through mechanisms still poorly understood. As a result, the level of highly reactive molecular species of oxygen (ROS) is likely to increase, with high potential to damage any biomolecule and activate even programmed cell death mechanisms; of special note are the damages that NPs can inflict on DNA and chromosome behavior. The typical cellular response is an increase of antioxidant enzymes (SOD, CAT, APX, *etc.*), so the changes in the cellular rate of ROS and anti-ROS and peroxidation products allow us to assess the degree of toxicity of NMs and the detoxifying ability of the plant cells. Oxidative stress induced by NMs can have long-term effects in some plant cells and in the case of upregulation of certain genes it can benefit plant growth and thus should be considered as potentially exploitable in agriculture [9]. Furthermore, it is known that the mitogen potentiality of ROS is able to induce DNA replication and cell proliferation; this could be a mechanism related to the increase in growth observed in some plants treated with NMs and therefore might be regarded as a benefit. Regardless, a number of favorable effects exploitable by man have been produced by NMs treatment [9] and perhaps major benefit for plants may come from NPs with their own antimicrobial and antifungal activity for use against pathogens [114,115].

When considering the reactive potential of NMs, several physicochemical traits should be emphasized without neglecting those acquired by residual impurities and exposure to air (airborne). Independently of the size, shape, surface structure, electronic structure, and charge, a basic characteristic is the nano-dimension, which allows a greater exposure of the atoms and a higher surface reactivity, albeit there is a wide range of reactivity and effects produced for the different types of nanoparticles. It can be expected that the smaller the surface area and the higher the zeta potential, the greater will be the reactive potential; there is no prediction about cell penetration and toxicity, especially as NPs are subject to various transformations and aggregation in the presence of natural organic matter [116]. Transformations include dissolution with release of metal ions, which are a factor of toxicity; chelation into complexes, which is within the detoxification mechanisms adopted by the plants; precipitation by chemicals arising from the growth environment or root secretion; and other reactions with numerous functional groups (hydroxyl, carboxyl, sulfhydryl, amino, *etc.*) that NPs encounter in the cellular pathways. In the case of CeO_2_–NPs, Cerium transforms into Ce^3+^ within the plant and in this form is considered toxic for cells [83]. Keeping in mind that Ce^3+^ tends to bind oxygen to return to Ce^4+^, we can hypothesize a switch of Cerium from 4+ to 3+ and the reverse in the cells, depending on the availability of oxygen; this could play a major role in oxidative stress. Conversely, in hydroponic cultures the ions of Ce^3+^ are precipitated in the roots by the phosphates that abound in the nutrient solution, and in the shoots are carboxylated by functional organic groups of the cells [48], producing unreactive forms. In addition to interactions of NPs with trophic and biological environments, we must not neglect those between different NPs hitherto known for Fe–NPs and Zn–NPs in the form of antagonism [23]. It is noteworthy that some metal ions within the plant are susceptible to transformation into NPs through redox reactions controlled by biomolecules, as recently reported by Kuppusamy *et al.* [117]. Hence, it is likely that within the plant a variable amount of metal ions resulting from NPs may again revert to NPs, in agreement with the previous observations of Larue *et al.* [25]. If the biosynthesis is to prevail to bio-dissolution, the NPs may concentrate and persist within the plant; their long-term effects are not known, while speculative applications regarding the biosynthesis of heavy and noble metal nanoparticles (gold, platinum, silver, zinc) are well known [117].

When comparing with animals, plant cells seem to be less damaged by NMs [7], and a role may be played by transmembrane transport via endocytosis, which is recurrent in animals—unlike in plant cells, where the cell wall and the pressure of turgor are an obstacle. Moreover, the large vacuolar system of plant cells may function as a detoxifying agent against NPs; the autophagy aforementioned [63] provides evidence. This activity, however, contrasts with the biosynthesis of NPs promoted by some vacuolar secondary metabolites [117]. It thus remains a matter of interest pending toxicological studies on differentiated cells (vacuolated) and undifferentiated cells (lacking vacuoles). An important point encompassing plant and animal cells is the potential of the plants to export NMs through their life cycle and the food chain, so, as already underlined by several authors [27,80], they could become carriers of chronic toxicity. Recent studies on watermelons and cucumbers [22,24] substantiate the ability of NMs to pass from the atmosphere into the leaf epidermis and its stomata, and then distribute inside the plant. Growing interest in the development of facilities for monitoring the impact of nano-materials on trophic and biological environments results from the self-sustaining mesocosm, which simulates natural ecosystems [118,119].

The emerging conclusions are that the studies on plants substantiate the reactive potential of nanomaterials; they are, however, based on different methods applied to different plant species, so the results lack homogeneity and are sometimes controversial and not ascribable to general models of interaction. A major limitation comes from the use of nanomaterials produced, characterized, and stored with different procedures that may confer different properties [120,121]; in particular, CNMs may retain active residuals of their precursors, such as Ni and Fe, and superficial pouches that expose the residuals. The methods of administration should ensure the control of the size and number concentration of NMs, as experienced by Wang *et al.* [22]. To shed light on the size limit for cell penetration, it is useful to know the sizes of NMs (hydrodynamic diameter) in the administration medium. The experiments with hydroponic plants and *in vitro* cell cultures, although they do not preserve the identity and integrity of the *in situ* plants, are useful and valuable for comparative purposes, not forgetting that adverse effects on plant cells may result from depletion of the micronutrients that interact with NMs. Furthermore, the techniques for detecting NMs within tissues and testing their toxicity to cells may produce artifacts, so special accuracy in preparing and manipulating NMs is needed in order to avoid misinterpretations; see [121].

The last point concerns the recurrent use of young plants, which limits the study to the vegetative phases of actively growing organs; it is hoped that studies will give more attention to life cycles and reproductive systems. The male and female generations producing gametes consist of a few cells with set haploid so they are easily vulnerable, especially during their pregamic interaction [122]. Closely related and to be evaluated thoroughly are the possible transgenerational effects of NMs.

While it remains important to study the basic mechanisms of the interaction of NMs with plants, our priority is to weigh up the potential benefits of NMs for plants and humans against the risk of exposing terrestrial ecosystems to particles that are small and potentially reactive.
